# Pancreatic Lymphatics in Health and Disease: Evolution, Embryology, and Neural Control

**DOI:** 10.3390/lymphatics4010001

**Published:** 2026-01-07

**Authors:** Alison Ross, Shakti Dahiya, Paulina Cabada Aguirre, Michael T. Lotze, Jami L. Saloman, Genia Dubrovsky

**Affiliations:** 1 Division of Surgical Oncology, Department of Surgery, University of Pittsburgh School of Medicine, Pittsburgh, PA 15213, USA; 2 Hillman Cancer Center, University of Pittsburgh, Pittsburgh, PA 15213, USA;; 3 Department of Medicine, Division of Gastroenterology, Hepatology, and Nutrition, University of Pittsburgh School of Medicine, Pittsburgh, PA 15213, USA; 4 Center for Neuroscience, University of Pittsburgh, Pittsburgh, PA 15213, USA; 5 Department of Immunology, University of Pittsburgh, Pittsburgh, PA 15213, USA; 6 Department of Bioengineering, Swanson School of Engineering, University of Pittsburgh, Pittsburgh, PA 15213, USA; 7 Division of Surgical Services, VA Pittsburgh Healthcare System, Pittsburgh, PA 15240, USA

**Keywords:** pancreas, pancreatitis, pancreatic ductal adenocarcinoma, lymphatics, innervation

## Abstract

Diseases of the pancreas—such as pancreatic ductal adenocarcinoma (PDAC) and pancreatitis—have long been a challenge to treat. The study of lymphatics within the pancreas can provide some additional insights and offer new therapeutic targets. Here, we explore the development of pancreatic lymphatics and their connections to the nervous system and individual disease states, as well as the potential for therapeutic interventions. Lymphangiogenesis pathways in PDAC, driven by VEGF-C and other mediators, have been extensively explored, but specific therapeutic interventions are lacking. Furthermore, due to the emergence of PDAC with pancreatitis, insights could improve treatment in both settings. The role of neuroimmune interactions and control, as in other organ sites, appears as critical to both lymphatic and immune processes. With a better understanding of the lymphatic environment within the pancreas, we can develop more effective treatments for patients.

## 1. Introduction

The lymphatic system plays a crucial role in the homeostasis of pancreatic function, as well as during both benign and malignant pancreatic pathophysiology. Importantly, there is evidence for adaptive and maladaptive functions in these perturbed states. For example, when the lymphatic system is compromised during an episode of acute pancreatitis, there is reduced clearance of damaging enzymes and thus an increase in tissue necrosis [1,2]. On the other hand, the lymphatic system has also been implicated as a vital route for distant cancer propagation via lymph node metastases [3]. Unfortunately, there is still a limited understanding of the mechanisms at play that determine lymphatic flow, lymphatic trafficking, and lymphatic remodeling, specifically with regard to the pancreas. In this review, we present the current understanding of the landscape of pancreatic lymphatics, highlighting the interplay with the peripheral nervous system and their individual roles in disease. This will provide novel avenues for potential therapeutics and areas of further study.

## 2. Early Development and Embryology of Pancreatic Lymphatics

Innate immunity developed over the last four billion years of life on earth [4,5]. Although the adaptive immune response emerged about 550 million years ago at about the same time that diffuse islet organs and the spleen developed in Agnatha (jawless fish) [6,7], the identification of a distinct pancreas could only be found later in jawed vertebrates such as sharks and other cartilaginous fish [8–10]. Adaptive immunity in Agnatha included a unique means of generating diversity with so-called VLRs (variable-like regions), distinct from the VDJC (variable, diversity, joining, constant) regions of modern T and B cells, which are similarly arranged [11–13]. Still, recognizable and distinct immune cell subsets could be easily identified and mapped onto soluble antibody-like molecule-secreting B cells, γδ T cells, CD4 and CD8 αβ T cells. Although non-mammalian species harbor diverse repertoires of T and B cells, they lack lymph nodes [14], which only emerged completely with mammals (n.b. ‘*Archicebus achilles*’ on the Yangtse River in China was the oldest identified primate, weighing 20 g during the Eocene Epoch, about 55 million years ago [15]). Interestingly, sharks have recently been observed to harbor germinal centers within the pancreas, located within distinct areas separate from the endocrine and exocrine pancreas and capable of secreting antibodies [16]. One can only presume that their disappearance during the emergence of lymph nodes draining the pancreas and other tissues was an ‘adaptive’ and relatively recent evolutionary development.

The first mammals are identified during the Late Triassic period, around 225 million years ago, when the supercontinent Pangea existed [17,18]. Thus, we maintain that the major role for adaptive immunity on the backdrop of 3.5 billion years of innate immunity was primarily to survey and respond to disorders of the host epithelium, including the pancreas. The implication of this speculative notion is that there is ‘reciprocal learning’ between the evolving pancreatic epithelium, as with other epithelia, and the local immune cells [19]. Recent studies suggest that inhibition of oncogenic KRAS in the setting of pancreatic cancer, for example, allows reemergence of such suppressed immunity [20].

The pancreas in humans can be visualized as early as gestational day 25–27 as transient notochord-dorsal pre-pancreatic endoderm contacts [21]. By day 30, two ventral buds are formed, which fuse at six to seven weeks of gestation. Acinar differentiation and islet vascularization initiate at 11 weeks, with ductal structures appearing at 24–32 weeks. Lymphatic Vessel Endothelial Hyaluronan Receptor 1 (LYVE1)-expressing lymphatics can be visualized around 9 weeks of gestation [22]. Activated pancreatic stellate cells limit CD8+ T cell migration [23] into the epithelium, allowing retention within the stroma.

## 3. Molecular Biology and Markers, Neuronal Control of the Pancreatic Lymphatics

### 3.1. Anatomy of Innervation

Neuroimmunology explores the bidirectional communication between the nervous and immune systems across development, homeostasis, and disease. While early work in this field centered on the central nervous system—particularly interactions between neurons and microglia—recent advances have expanded our understanding of neuroimmune dynamics in peripheral tissues, including the pancreatic lymphatics [24–26]. Neuroimmune crosstalk emerges during development and continues through adulthood, regulating maturation, priming, and proliferation of resident and recruited immune cells [27–29]. Recent studies explore molecular neuroimmune mechanisms identified during development. Clustering of hematopoietic lymphoid tissue-inducer (LTi) cells with stromal organizer cells is one of the first steps in lymph node (LN) development. The clustering of these cells requires the expression of cytokines, such as CXC-chemokine ligand 13 (CXCL13), which is induced by nerve-derived retinoic acid, provocatively suggesting a role of the peripheral nervous system early in LN development [30,31].

**Figure 1.**
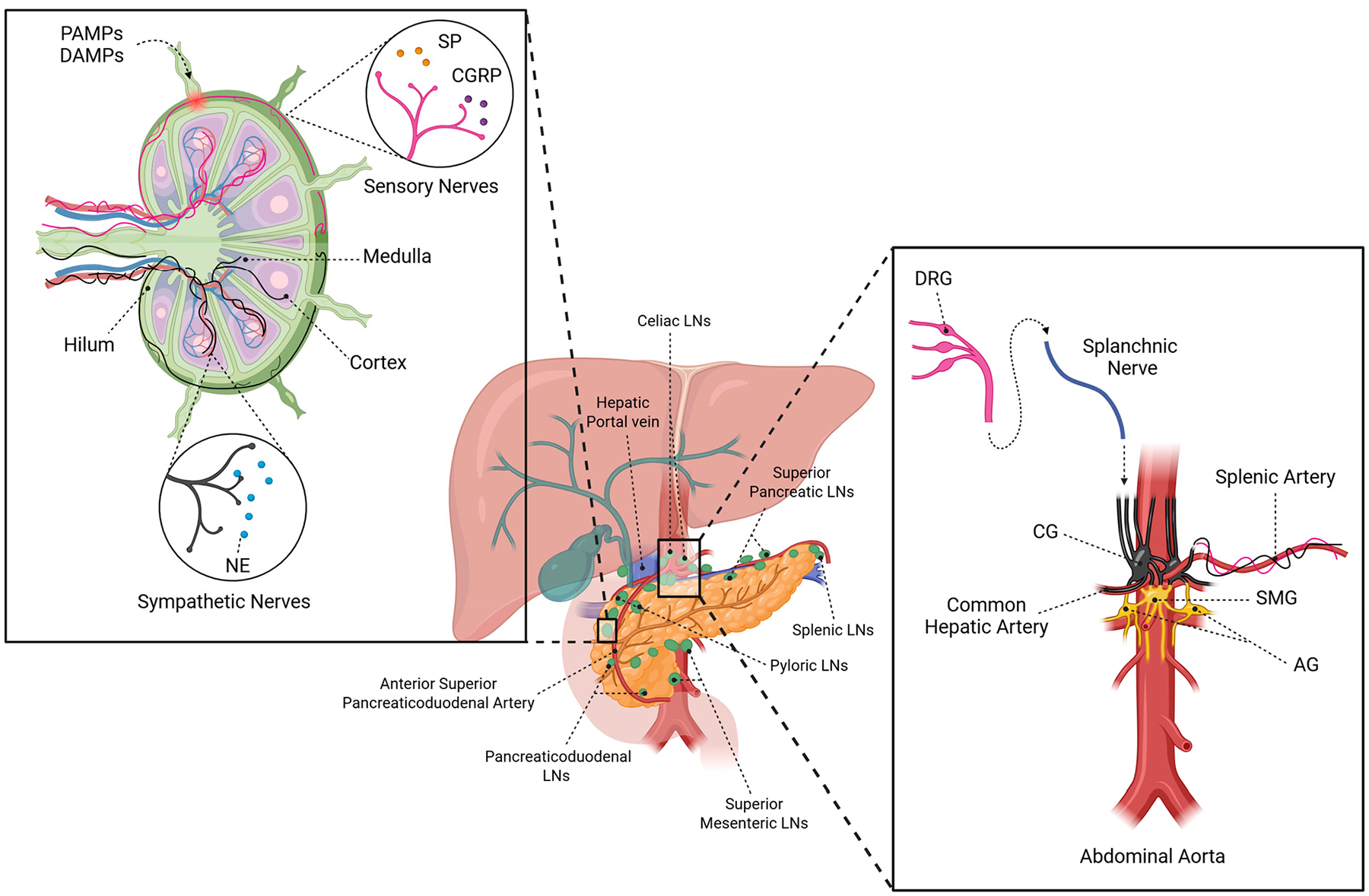
Anatomy of Pancreatic Lymph Nodes and their Innervation. Simplified schematic representation of the neural circuits innervating pancreatic lymph nodes. Sympathetic fibers (black) reach the lymph node via the celiac ganglion (CG) and splanchnic nerve, releasing norepinephrine (NE) and peptides within the node. Paravascular and distinct sympathetic fibers are distributed across the hilum, capsule, cortex, and medulla. Sensory nerves (pink) originate from dorsal root ganglia (DRG) and travel through the splanchnic nerve to reach the pancreatic lymph nodes. These nerve fibers release glutamate and neuropeptides such as substance P (SP) and calcitonin gene–related peptide (CGRP) in response to stimuli such as pathogen-associated molecular pattern molecules (PAMPs) and damage-associated molecular pattern molecules (DAMPs). Paravascular sensory nerve bundles, as well as distinct fibers, terminate in the capsule, tubercule, parenchyma, and cortex. The central schematic highlights the anatomical relationship of pancreatic lymph nodes (green) with the pancreas, liver, and major abdominal vasculature. SMG Superior Mesenteric Ganglia; AG Aortic Renal Ganglia.

Anatomical studies of the peripheral nervous system (PNS) in multiple human and animal models report sensory and sympathetic innervation with minimal parasympathetic input of LNs and lymph vessels [32–41]. A schematic representation of the peri-pancreatic lymph nodes and associated neuronal fibers is shown in Figure 1. The peri-pancreatic lymph nodes have been carefully categorized by their anatomic relationships to the pancreas, surrounding blood vessels, and bile duct [42]. Specifically for pancreatic LNs within human fetuses, lymphatic drainage is organized in parallel to nerve fibers emanating from the celiac ganglia. A high density of capillaries and lymphatic vessels can be identified starting at the origin of the celiac trunk and continuing along the hepatic and splenic arterial branches along with nerve bundles [43]. While some microanatomical studies have observed a dense neural network with nerve endings penetrating all compartments, including B cell follicles and germinal centers [38], others report no innervation of B cell follicles and germinal centers, along with an overall lower density of fibers [44]. Disagreements between studies may be attributed to the use of varied techniques. One study used cleared tissue that was immunolabeled with neuronal markers, such as PGP9.5 (a pan-neuronal marker), tyrosine hydroxylase (TH), and vesicular acetylcholine transporter. The second study used anterograde labeling, injecting Fluoro-Ruby dye into the superior cervical ganglion and capturing only sympathetic fibers emerging from this ganglion, excluding sympathetic fibers from other origins as well as sensory fibers captured with an immunohistochemical approach [38,44].

Immunohistochemical staining with PGP9.5 in adult human and non-human primate LNs reveals an irregular innervation pattern. In humans, nerve fibers are observed mostly in the tissue surface, while in rhesus macaques, fibers penetrate the parenchyma. Both tyrosine hydroxylase (TH) and vesicular acetylcholine transporter-positive fibers can be identified. This confirms the presence of sympathetic fibers and cholinergic fibers identified as parasympathetic [38]. However, TH is not specific for sympathetic fibers, nor are all cholinergic fibers parasympathetic. Tracing studies are needed to determine the origin and classification of the observed fibers [45–47]. Additionally, findings regarding expressions of sensory fiber markers, such as CGRP, are lacking. Reports in rodent and canine models suggest that sensory innervation modulates LN activity. Still, there is a gap in knowledge concerning human LN innervation that needs to be further studied [35,37,41].

The presence of both myelinated and unmyelinated fibers has been reported in axillary, popliteal, and inguinal rodent LNs. Studies characterizing LN innervation through electron microscopy and immunohistochemistry staining for neurofilament heavy chain (NFH) have established a higher abundance of unmyelinated fibers [41,48,49]. Myelinating and non-myelinating Schwann cells (MSCs and NMSCs, respectively) also contribute to immune modulation in the periphery through multiple mechanisms, including the expression of major histocompatibility complex (MHC) class II molecules, which are usually expressed by antigen-presenting cells, including macrophages, dendritic cells (DCs), and B lymphocytes and most other cells only after stimulation with interferon gamma. MHC Class II molecule expression, shown in human and rodent models of immune-mediated disorders of the peripheral nervous system such as Guillain–Barré syndrome, suggests a possible role of Schwann cells as putative antigen-presenting cells. A recent study in healthy murine mesenteric LNs showed close interactions between NMSCs and two macrophage subsets (CD11b^+^ and F4/80^+^), influencing macrophage antigen presentation and cytokine production. They also showed evidence of NMSCs’ homeostatic interactions with multiple subsets of dendritic cells, lymphatic vessels, and capillaries [27]. To our knowledge there are no pancreatic LN-specific data demonstrating a role and enumeration of myelinated and unmyelinated axons or the role of Schwann cells in modulating pancreatic immune responses.

### 3.2. Sympathetic Innervation

Sympathetic nerves innervate LNs through two primary structural patterns. Paravascular nerve bundles follow blood vessels into LN compartments, while discrete nerves branch independently into parenchymal regions, including T-cell zones in the cortex and medulla [26,34]. In human LNs, sympathetic fibers are consistently present in the capsule, medulla, and hilum, while cortical innervation is variable. Varicosities—indicative of neurotransmitter release sites—are observed along these fibers, confirming functional synaptic capacity [34]. Studies focused on pancreatic LNs demonstrated receipt of sympathetic input via mesenteric nerves, which also innervate intrapancreatic ganglia, islets, ducts, and vasculature [50]. Innervation originates from celiac ganglia and splanchnic nerves [25,50].

Sympathetic neurons innervating the LN can locally release neurotransmitters that modulate immune activity. The main sympathetic neurotransmitter, norepinephrine (NE), affects immune responses by binding to adrenergic receptors on immune cells. This engagement can alter cytokine production, antigen presentation, and cell migration [51–54]. NE release can be increased in the context of disease due to chronic stress. This results in remodeling LN innervation, increasing adrenergic nerve density in follicular regions [34,55]. In cancer models, settings in which chronic stress is present, NE increases lymphatic vessel density and diameter via β-adrenergic receptors. Whether they regulate lymphatic valves is unclear [56–63]. This, in turn, promotes an increase in lymphatic flow, providing a route for cancer cell dissemination and metastasis [64]. This is consistent with literature showing β-adrenergic receptor agonists induce nitric oxide-dependent vasodilation. Additionally, tumor-draining lymphatic vessels demonstrate higher innervation density and contractility that can be reduced through treatment with isoproterenol. It is important to note that this study found differential tissue-specific expression of muscarinic and adrenergic receptors in skin and intestinal draining lymphatic vessels, limiting the translation of these findings into pancreatic draining lymphatics [65]. The increased sympathetic innervation observed in primate LNs under chronic stress conditions results in the suppression of IFN-β while simultaneously upregulating IFN-α production, representing an additional mechanism of sympathetic immune modulation. Type one interferon (IFN-β and IFN-α) signaling is crucial for immune activation and can be shifted by changes in LN sympathetic neural architecture to result in an overall immunosuppressive state [52,55].

### 3.3. Role in Autoimmunity

Sympathetic signaling also modulates autoimmunity mechanisms within secondary lymphoid structures. In a non-obese diabetic (NOD) mouse model of spontaneous type 1 diabetes (T1D), sympathetic activation in pancreatic LNs, through neural stimulation, inhibited disease progression. This occurred via β-adrenergic receptor-mediated accumulation of B and T cells and reduced proliferation of autoreactive T cells in pancreatic LNs. The stimulated nerve was resected, and immunohistochemical staining with TH showed a significant decrease in sympathetic nerve fibers in the pancreatic draining LNs but not in the pancreas. This confirms the observed changes in immune populations after electrical stimulation are due to changes in neural activity localized to the LNs and not the nerves innervating the pancreatic islets [39]. This is further supported by T1D exacerbation due to the selective inhibition of β-adrenergic receptor signaling with propranolol in the RIP-LCMV-GP mouse model of autoimmune diabetes. Conversely, systemic administration of an α-adrenergic receptor inhibitor provided a protective effect from T1D [66]. The specific role of α-adrenergic signaling has not yet been studied in pancreatic lymphatics.

### 3.4. Sensory Innervation

Spinal sensory afferent nerve fibers arise in dorsal root ganglia (DRG) and travel alongside sympathetic fibers in the splanchnic nerves to innervate pancreatic LNs. Spinal sensory fibers pass through the celiac ganglion, which harbors no parasympathetic fibers. Parasympathetic and sensory fibers arising from the nodose ganglia travel in the vagus nerve, but it is not clear that these innervate LNs.

Sensory fibers have been mapped within the capsule, trabeculae, and parenchyma of mammalian LNs [32,34,40]. Although not uniform across all LNs or nodal compartments, in both mice and humans, peptidergic sensory fibers are most prominent in the capsule and medullary regions but can also be found in the cortex and paracortex [41]. Sensory fibers innervating LNs include unmyelinated and lightly myelinated Aδ axons [48]. These fibers are often present near arterioles and venules in the hilus, medullary region, and internodular region. Sensory nerves have also been observed in proximity to lymphatic endothelial cells and specific immune cell populations such as dendritic cells and macrophages [35,41,48,67]. In canine mesenteric LNs, receptor binding sites for sensory neuropeptides are present in both the capsule and parenchyma, suggesting potential for direct neuroimmune signaling [35].

Activation of sensory neurons by inflammatory mediators, pathogen/damage-associated molecular pattern molecules (PAMPs/DAMPs), or other mediators of tissue injury [68] induces release of neuropeptides such as Calcitonin Gene-related Peptide (CGRP) and Substance P (SP). Characterization of peptidergic fibers in mesenteric, axillary, and inguinal lymph nodes obtained from rats, mice, guinea pigs, and humans determined that fibers expressing both SP and CGRP were the most abundant in both vascular and lymphatic compartments [37,69]. Additionally, sensory fibers co-express SP with dynorphins or cholecystokinin and localize only to the hilus and medulla. These findings suggest the existence of distinct sensory neuron populations within the lymphatic tissue and LN-associated vasculature [37]. This is in agreement with more recent reports of sensory innervation architecture in the LN being organized into two nerve plexuses, one perivascular and another capsular/subcapsular [41]. These neuropeptides can directly influence dendritic cell (DC) maturation, T cell activation, and cytokine production. Studies in mammalian models have shown LN-innervating sensory neurons play a role in capsaicin (TRPV1 agonist)-induced inflammatory responses, antigen retention, and flow through peripheral LNs [41,70,71].

LNs are innervated by a molecularly distinct population of sensory neurons than those targeting the skin or visceral organs. Nerve endings from these neurons physically associate with LN immune cell populations, particularly in T cell zones, causing immune suppression through CGRP activity on DC. Immunosuppression can be reversed through CGRP blockade, suggesting it has a major role in modulation of the LN immune response [41]. Another example of sensory nerve-driven immune modulation is the systemic ablation of Na_v_1.8 lineage nociceptor neurons before skin bacterial infection, which results in an absence of pain while concurrently increasing immune influx and lymphadenopathy [72]. Conversely, nociceptor ablation in an imiquimod-induced psoriasis model had the opposite effect by reducing hypertrophy of regional LNs and skin inflammatory response [73]. Altogether these results suggest sensory neurons innervate LNs across the body and have a context-dependent effect on immune responses. The mechanisms by which these context-dependent effects occur in the pancreas have yet to be studied and represent an area of opportunity for investigation in the field.

### 3.5. Development of Tertiary Lymphoid Structures

The term tertiary lymphoid structure (TLS) refers to lymphoid aggregates often formed due to chronic inflammation within non-hematopoietic organs. Like the development of secondary lymphoid organs, TLS depend on interactions between lymphoid tissue inducer cells (LTis) and organizer cells induced by multiple cytokines [74,75]. TLS have been found within both intra- and peri-tumoral tissues in pancreatic cancer. A retrospective human study found colocalization of small nerve fibers as well as lymphoid aggregates in pancreatic cancer tumor samples. In patients with five or more TLS, a higher density of nerve fibers correlated with a better prognosis [76]. It is unclear how this relates to the observations that hyperinnervation and perineural invasion within the pancreatic tumor microenvironment (TME) correlate with worse prognosis. Nonetheless, the extent to which the nervous system impacts the development of pancreatic TLS is still unclear. In other disease models, such as melanoma, the presence of sensory nerves impedes the formation of TLS. Chemical sensory denervation results in smaller intratumoral populations of Treg and Gr1^+^ neutrophils, which inhibit TLS formation [77]. On the other hand, chemical ablation of sympathetic innervation results in a significant decrease in TLS density and number following acute pulmonary inflammation induced through intranasal lipopolysaccharide administration [77].

Despite significant advances in mapping the neural anatomy of LNs and elucidating the neuroimmune mechanisms at play, substantial gaps remain—particularly regarding the sensory and sympathetic innervation of pancreatic LNs. Most foundational studies have focused on LNs in general or on non-pancreatic sites, with only a handful of anatomical investigations specifically addressing the unique features of pancreatic LNs. The available data, often derived from fetal or animal models, suggest that pancreatic LNs are richly innervated, yet the precise density, distribution, and functional specialization of sensory and sympathetic fibers (traveling in the splanchnic nerves) in adult human pancreatic LNs remain poorly characterized. While sympathetic modulation of immune activity in LNs has been increasingly studied in the context of stress, infection, and autoimmunity, the specific contributions of sensory innervation—especially in pancreatic LNs—are only beginning to be appreciated. The unique neuroimmune environment of the pancreas, coupled with its relevance to diseases such as pancreatitis, type one diabetes (T1D), and pancreatic cancer, suggests a need for targeted research in this area.

## 4. Pancreatic Lymphatics in Pancreatitis

Characterized as inflammation of the pancreas, there are many forms of pancreatitis, generally grouped into acute (AP), recurrent acute (RAP), or chronic (CP) pancreatitis [78]. Lymphatics, as a major component of the immune system, contribute to the resulting inflammation but also present a potential therapeutic target [79]. Several innate immune cells have demonstrated involvement in pancreatitis, such as neutrophilic granulocytes, monocytes/macrophages, and pancreatic acinar cells (Table 1) [79–83].

### Pancreatitis and Acinar Cells

Pancreatic acinar cells are the primary pancreatic cells damaged in pancreatitis [78]. This damage results from an inflammatory response to an initial pancreatic injury (in AP), which can progress to CP [84]. During acute injury, the pancreatic acinar cells hyper-secrete proteases as well as inflammatory molecules such as NFκB, TNF, IL-4, IL-6, IL-10, and MCP-1, which lead to further pancreatic inflammation with recruitment of neutrophils and monocytes/macrophages [79,82,83]. Once activated within the pancreas, the neutrophils release proteases and reactive oxygen species (ROS), promoting pancreatic cell death [79,80]. Monocytes/macrophages can also promote inflammation by releasing additional proinflammatory molecules (TNF, IL-6, and IL1β) [79,81]. These molecules in turn regulate lymphatic flow [85].

Recent investigations utilizing transcriptomics are beginning to reveal cell-state characteristics and targetable pathways in pancreatitis (Table 2) [86,87]. Common pathways include those involved in oxidative phosphorylation, apoptosis, NF-κB signaling, TNF release, and interleukin production [86,87].

## 5. Pancreatic Lymphatics in Cancer

### 5.1. Lymphangiogenesis and PDAC Progression

Lymphangiogenesis, the process by which new lymphatic vessels sprout from existing vasculature, is a pivotal driver of pancreatic ductal adenocarcinoma (PDAC) progression. Among the lymphangiogenic signaling axes, the vascular endothelial growth factor (VEGF) family is the most extensively characterized. VEGF-C and VEGF-D can interact with VEGFR-3 to promote lymphangiogenesis. These cytokines facilitate the proliferation, migration, and remodeling of lymphatic endothelial cells (LECs) and can contribute to tumor dissemination [89].

The accurate identification of lymphatic vessels is essential for assessing lymphangiogenesis in pancreatic cancer tissues. Several histological markers, such as LYVE-1, Prox1, and podoplanin (D2–40), are widely used in immunohistochemistry and in situ imaging to delineate lymphatic endothelium in both animal models and human samples. These markers help differentiate lymphatics from blood vessels and have been instrumental in correlating lymphatic vessel density (LVD) with clinical outcomes in PDAC. A comprehensive summary of these markers is presented in Table 3.

### 5.2. Cytokines and Growth Factors Associated with Lymphangiogenesis in PDAC

Cytokines and growth factors play a central role in orchestrating lymphangiogenesis within the pancreatic tumor microenvironment.

#### VEGF-C.

VEGF-C is the most extensively characterized and is frequently upregulated in PDAC, where it directly correlates with lymphatic invasion and nodal metastasis. Several regulatory mechanisms of VEGF-C have been elucidated in recent years. A novel DUSP2–VEGF-C–EV signaling axis, with loss of the tumor suppressor DUSP2, promotes ERK activation and enhances EV-mediated secretion of bioactive VEGF-C [89]. These vesicles, in turn, stimulate lymphatic endothelial proliferation and promote both lymphangiogenesis and tumor cell invasiveness. Pharmacologic restoration of DUSP2 expression using a selective HDAC1/2 inhibitor (B390) leads to significant reductions in VEGF-C levels, lymphatic vessel density, and lymphovascular invasion in orthotopic mouse models, highlighting this pathway as a potential therapeutic target [98]. Extending this axis into the inflammatory tumor microenvironment, tumor-associated macrophages are key drivers of DUSP2 suppression through secretion of TIMP-1. Macrophage-derived TIMP-1 binds CD63 on cancer cells to prolong ERK phosphorylation, reinforcing a stable ERK^active^/DUSP2^low^ state. These cancer cells subsequently upregulate production of VEGF-C and PD-L1 expression, promoting both lymphangiogenesis and immune evasion. Spatial transcriptomics of human PDAC confirms colocalization of TIMP-1^high^ macrophages and CD63^high^ cancer cells, with this phenotype correlating with aggressive lymphatic invasion, advanced disease, and poor patient survival. Notably, DUSP2 knockdown or macrophage co-culture recapitulated these effects, highlighting the ERK^active^/DUSP2^low^ axis as a convergent point of tumor-intrinsic and immune-mediated signaling. Collectively, these studies underscore the dual role of the ERK–DUSP2–VEGF-C pathway in coordinating structural lymphangiogenesis and immune escape, revealing it as a promising therapeutic target in KRAS-mutated PDAC [99].

Several preclinical studies support this mechanism. VEGF-C and VEGF-D expression levels significantly correlate with lymph node metastasis and reduced overall survival in PDAC patients [100]. VEGF-C-rich tumors induce both peritumoral and intratumoral lymphangiogenesis, marked by elevated LEC proliferation. These findings reinforce the pivotal role of VEGF-C/VEGF-D–VEGFR-3 signaling in establishing a lymphatic niche conducive to metastatic spread [101].

#### Angiopoietin-2.

In addition to VEGF-C, Angiopoietin-2 (Ang-2) is a potent pro-lymphangiogenic and pro-metastatic cytokine in PDAC. Ang-2, signaling via its receptors Tie1 and Tie2, plays pivotal roles in lymphatic development and immune-endothelial interactions. VEGF-C promotes Ang-2 secretion from lymphatic endothelial cells (LECs), which subsequently enhances PI3K/Akt signaling and facilitates VEGFR3 membrane localization. These are both essential for LEC responsiveness and lymphatic sprouting. Genetic ablation of Ang-2, Tie receptors, or PI3K subunits disrupts this feedback loop, leading to impaired lymphangiogenesis and reduced metastatic dissemination in PDAC models [102]. Supporting this mechanism in a clinical context, Ang-2 is de novo expressed in human PDAC tissues. Its circulating levels strongly correlate with lymph node metastasis and poor prognosis. Patients with Ang-2 levels in the upper quartile have markedly reduced survival compared to those with lower levels. Together, these findings highlight the Ang-2/Tie2/PI3K axis as a central conduit linking inflammatory signaling to lymphatic remodeling and metastatic spread in PDAC, and they underscore the translational potential of targeting this pathway to curb lymphatic dissemination [103].

### 5.3. Lymphangiogenesis and Mutant KRAS

A VEGF-C–independent mechanism is promoted by KRAS^G12X^ mutations, which are found in over 90% of PDAC cases. This can drive lymphangiogenesis and lymph node (LN) metastasis [104]. Mutant *KRAS*^*G12D*^ upregulates SUMO-activating enzyme subunit 1 (SAE1), leading to SUMOylation of the RNA-binding protein hnRNPA1 at lysine 113. This post-translational modification facilitates its interaction with TSG101, promoting its selective packaging into extracellular vesicles (EVs). These EVs are then internalized by human lymphatic endothelial cells (HLECs), where SUMOylated hnRNPA1 binds to AU-rich elements within the 3′ untranslated region of PROX1 mRNA, stabilizing it and enhancing PROX1 expression. PROX1 plays a major role in lymphatic differentiation and development. Functionally, EV-packaged hnRNPA1 increases tube formation and migration of HLECs in vitro and promotes LN metastasis in vivo, as shown in both popliteal and orthotopic xenograft models. Importantly, these effects are reversed by disrupting hnRNPA1 SUMOylation or silencing PROX1, confirming the essential role of this axis. This KRAS-driven mechanism induces robust lymphatic remodeling and LN metastasis independent of VEGF-C signaling. hnRNPA1 expression positively correlates with micro-lymphatic vessel density and LN involvement in PDAC tumors. These findings establish EV-mediated, SUMOylation-dependent hnRNPA1 transmission as a novel driver of lymphangiogenesis and a potential therapeutic target in KRAS-mutant PDAC.

Earlier studies have extensively characterized animal models of PDAC progression and metastasis, particularly *KRAS*^*G12D*^-based genetically engineered mouse models (e.g., KPC, KIC), which faithfully recapitulate local invasion and lymphatic spread [105,106]. These models provide a valuable framework for validating novel mechanisms, such as the KRAS–SUMO–EV–PROX1 axis, and for developing targeted interventions against lymphatic dissemination.

### 5.4. Targeting Lymphatics in PDAC

Using an orthotopic mouse model, short hairpin RNA (shRNA) targeting VEGF-C reduced tumor growth and markedly suppressed micro-lymphatic vessel density (MLVD) [107]. Interestingly, while gemcitabine more effectively reduced tumor volume, VEGF-C shRNA exerts superior inhibitory effects on lymphangiogenesis, confirming VEGF-C’s central role in lymphatic remodeling and metastasis. Non-coding RNAs also regulate inflammatory signaling and lymphangiogenesis in PDAC. circNFIB1 is a tumor suppressor that sequesters miR-486–5p, thereby upregulating PIK3R1, a negative regulator of the PI3K/Akt pathway [108]. Suppression of PI3K signaling reduces VEGF-C expression, leading to decreased lymphatic vessel formation and lymph node metastasis in vivo, highlighting the role of circular RNAs in modulating pro-lymphangiogenic signaling. TGF-β1 and GM-CSF also promote lymphangiogenesis [109]. Their expression correlates with M2-type tumor-associated macrophages (TAMs) and increased MLVD, particularly following chemotherapy. Dual blockade of TGF-β1 and GM-CSF not only enhanced gemcitabine efficacy but also reduced lymphatic vessel density and promoted immune activation, indicating that targeting inflammatory cytokines could disrupt pro-lymphangiogenic conditions.

### 5.5. Targeting Signaling Pathways in Lymphatics

#### PI3K/Akt/mTOR.

The PI3K/Akt/mTOR signaling cascade is a central pathway in VEGF-C regulation. Rapamycin, an mTOR inhibitor, significantly decreases VEGF-C expression and lymphangiogenesis in a lymphatic metastasis-prone PDAC model [110]. This effect is observed both at the transcriptional level and in xenograft models, establishing mTOR signaling as another viable therapeutic mode to restrict early lymphatic dissemination. Together, these findings position cytokine and growth factor signaling as central regulators of lymphangiogenesis in PDAC. Pathways converging on VEGF-C expression are promising targets for anti-metastatic interventions.

#### Other Cytokines.

In addition to VEGF-C, proinflammatory cytokines such as IL-8, CXCL1, and CCL2 are upregulated in the PDAC microenvironment and contribute to lymphangiogenesis. miR-206, a tumor-suppressive microRNA, downregulates these cytokines/chemokines by targeting KRAS and ANXA2, with consequent inhibition of NF-κB signaling [111]. Restoration of miR-206 in PDAC models led to reduced VEGF-C expression, diminished LYVE-1+ lymphatic vessel density, and decreased lymphatic dissemination. These findings suggest that miR-206 not only suppresses tumor growth but also attenuates inflammatory lymphangiogenesis, making it a promising candidate for RNA-based therapeutic intervention.

### 5.6. Inflammatory Cell Subsets Involved in Lymphangiogenesis

Inflammatory and stromal cells within the TME actively contribute to lymphangiogenesis by secreting pro-lymphangiogenic mediators and engaging in direct interactions with lymphatic endothelial cells (LECs). Among the critical mediators in this context is sphingosine phosphate kinase 1 (SPHK1), an enzyme that produces sphingosine-1-phosphate (S1P). S1P is a bioactive lipid that regulates immune cell trafficking, endothelial permeability, and angiogenesis. In pancreatic cancer, SPHK1 is frequently overexpressed and correlates with increased lymphatic vessel density (LVD) and lymphatic invasion. Silencing SPHK1 in pancreatic cancer cells markedly impairs LEC tube formation in vitro. Mechanistically, SPHK1 activates the ERK1/2 signaling pathway, which is essential for LEC proliferation and vessel sprouting. These findings implicate SPHK1 not only as a metabolic regulator but also as a mediator of inflammatory signaling that facilitates lymphatic remodeling and metastasis [112].

These studies underscore how inflammatory cell–driven signaling cascades, including the SPHK1–ERK1/2 and Ang2–Tie–PI3K–VEGFR3 pathways, shape the lymphatic niche and contribute to tumor progression. Targeting these axes may offer promising avenues for curbing lymphatic metastasis in PDAC. Multiple cytokines, growth factors, and non-coding RNAs influence lymphangiogenesis through distinct molecular mechanisms. A detailed summary of these mediators and their roles in PDAC is presented in Table 4.

### 5.7. Immune Cell Plasticity and Tumor-Intrinsic Contributions

Beyond soluble factors, the immune system contributes directly to lymphangiogenesis through non-classical mechanisms. Tumor-associated macrophages (TAMs) in PDAC can also express LYVE-1 and VEGFR-3, suggesting their ability to transdifferentiate into lymphatic-like cells. This process implies that immune cells not only regulate but may structurally contribute to lymphatic vessel formation, adding another layer of complexity to the metastatic cascade [122].

Further reinforcing the immuno-lymphatic interface, FOXP3^+^ regulatory T cells (Tregs) are also associated with increased CCL21 expression in peritumoral lymphatics, correlating with lymph node metastasis [123]. These findings support a feedback loop in which immunosuppressive cell populations and chemokines co-evolve to sustain and expand metastatic lymphatic niches. PDAC cells can also directly stimulate LEC proliferation and migration by secreting lymphangiogenic cytokines, independently of immune cell involvement [124]. This underscores the autonomous capacity of tumor cells to remodel their lymphatic microenvironment. Taken together, these studies illustrate that immune cell plasticity, chemokine signaling, and tumor-secreted factors converge to shape a highly dynamic and metastatic lymphatic landscape in PDAC.

### 5.8. Non-Coding RNAs and Exosome-Mediated Regulation

Further emphasizing the role of tumor-derived exosomes, EVs from pancreatic cancer cells promote lymphangiogenesis by suppressing ABHD11-AS1, a long non-coding RNA that normally inhibits LEC activation. Loss of ABHD11-AS1 within LECs enhances their proliferation, migration, and tube formation, indicating that exosomal RNA cargo can directly reprogram the lymphatic endothelium. Restoring ABHD11-AS1 expression or preventing its suppression could offer novel anti-lymphangiogenic strategies [119].

Complementing these findings, long non-coding RNA BANCR also acts as a potent driver of lymphangiogenesis in PDAC. BANCR functions by sequestering miR-143–5p, leading to stabilization and upregulation of HIF-1α, a hypoxia-responsive transcription factor that drives VEGF-C/VEGFR-3 signaling. This axis increases micro-lymphatic vessel density (MLVD) and facilitates lymph node metastasis. BANCR knockdown significantly reduced MLVD and downregulated VEGF-C and VEGFR-3 expression, positioning it as both a prognostic biomarker and a potential therapeutic target [120].

Collectively, these findings underscore the expanding importance of non-coding RNA networks and exosome-mediated communication in regulating PDAC lymphangiogenesis. These pathways present mechanistically rich and clinically relevant avenues for future therapeutic development.

## 6. Inflammation-Driven Lymphangiogenesis

Lymphangiogenesis, often considered a hallmark of cancer progression, is increasingly recognized as a dynamic response to benign inflammation, including chronic pancreatitis, autoimmune infiltration, and pre-malignant pancreatic injury. This inflammatory lymphatic remodeling may establish a pre-metastatic niche and prime the TME for later cancer dissemination.

### 6.1. Lymphangiogenesis in Chronic Pancreatitis and Pre-Malignant Lesions

Chronic pancreatitis, a major risk factor for PDAC, exhibits histologic and molecular parallels with malignant disease. LVD in CP is comparable to that found in PDAC, particularly in the peritumoral regions [125]. Surprisingly, intratumoral LVD is lower in PDAC, suggesting that pre-existing lymphatic vessels formed during chronic inflammation may serve as conduits for early metastatic spread, rather than new vessels formed by the tumor itself. Extending this observation to the pre-malignant setting, 3D tissue-clearing and light-sheet imaging in *KrasG12D* and *KrasG12D*/p53+/− mouse models demonstrate marked lymphangiogenesis around PanIN lesions, characterized by endothelial invagination, lymphatic sprouting, and vessel dilation. These changes occur prior to the emergence of invasive carcinoma. Similarly, in cerulein-induced pancreatitis, robust lymphangiogenesis is detected near ducts and lymph nodes in the absence of neoplastic changes. These findings demonstrate that benign inflammatory cues alone can trigger lymphatic remodeling, likely via shared signaling axes that are later co-opted by tumors [126].

### 6.2. Immune-Associated Lymphangiogenesis in Inflammatory Pancreatic Conditions

Inflammatory conditions can induce lymphangiogenesis through immune-mediated mechanisms, including immune cell recruitment and cytokine signaling [127]. In a mouse model of autoimmune insulitis, lymphatic expansion around inflamed islets facilitates immune cell trafficking. Importantly, blocking VEGFR-3 signaling attenuated lymphangiogenesis, reduced T-cell infiltration, and preserved tissue structure, confirming that lymphatics are both responders and amplifiers of inflammation in non-malignant pancreatic disease [127]. Similarly, in the context of acute pancreatic inflammation, immune cell infiltration is accompanied by upregulation of lymphangiogenic factors, including VEGF-C and VEGFR-3 [128]. Their findings indicate that pancreatic injury, even in the absence of malignancy, activates molecular pathways that drive lymphatic vessel remodeling, contributing to stromal reprogramming and fibrosis [126].

### 6.3. Mechanistic Insights from Inflammation-Associated Lymphangiogenesis

Inflammation-associated lymphangiogenesis is a conserved response across multiple tissues and inflammatory contexts [129]. Cytokines such as IL-1β, TNF, and IFN-γ promote VEGF-C/D expression, while transcription factors such as NF-κB and Prox1 coordinate LEC proliferation, migration, and lymphangiogenic sprouting. These mechanisms are active in sterile inflammation, wound healing, and tumor-adjacent tissues, highlighting the shared biology between benign and malignant lymphangiogenesis [126].

## 7. Clinical Relevance of Lymphangiogenesis in PDAC

Lymphangiogenesis plays a pivotal role in the clinical progression and metastatic behavior of PDAC, representing a major pathway for tumor cell dissemination. Several clinical and translational studies have demonstrated that enhanced lymphatic vessel formation, particularly at the tumor periphery, strongly correlates with aggressive disease features and poor prognosis.

In 70 patients with pancreatic head cancer, increased LVD at the tumor margin was significantly associated with a higher incidence of lymph node metastasis and reduced overall survival. Strikingly, even non-metastatic lymph nodes in patients with node-positive disease displayed elevated LVD, suggesting that lymphangiogenesis precedes tumor cell arrival, potentially preparing a “fertile ground” for metastatic colonization as a premetastatic niche. This phenomenon of intranodal pre-metastatic lymphangiogenesis also correlates with metastasis size and para-aortic lymph node involvement, positioning LVD as both a prognostic and predictive biomarker for disease progression in PDAC [130].

When comparing peri-tumoral (pLVD) and intra-tumoral (iLVD) lymphatic vessel densities across pancreatic tumor types, iLVD did not correlate with clinical outcomes. Interestingly, pLVD was significantly associated with poor tumor differentiation, increased lymphatic invasion, and lymph node metastasis in PDAC [131]. Moreover, the pLVD level was markedly higher in malignant PDAC compared to benign or borderline tumors such as cystadenomas and pseudopapillary neoplasms, underscoring its role as a meaningful prognostic marker and indicator of malignant potential. These findings highlight the importance of assessing lymphatic remodeling at the invasive front rather than the tumor core for predicting disease behavior.

Collectively, these clinical and mechanistic studies establish lymphangiogenesis, especially at the tumor margins and within draining lymph nodes, as a clinically relevant biomarker of prognosis and a key facilitator of metastatic progression in PDAC. Therapeutic strategies aimed at disrupting lymphangiogenic signaling or targeting supportive immune subsets may hold promise for improving outcomes in patients with pancreatic cancer.

## 8. Therapeutic Strategies for Targeting the Pancreatic Lymphatics

Targeting pancreatic lymphangiogenesis represents a promising therapeutic frontier in reducing metastasis and improving outcomes in PDAC. Multiple strategies are under investigation, ranging from direct inhibition of lymphangiogenic growth factors to reprogramming the lymphatic niche and enhancing immunotherapy responsiveness.

### 8.1. Inhibition of Canonical Lymphangiogenic Pathways

The VEGF-C/VEGFR-3 signaling axis is the most extensively studied and clinically relevant pathway driving lymphangiogenesis in PDAC. VEGF-C, secreted by both tumor cells and tumor-associated macrophages (TAMs), binds to VEGFR-3 receptors on LECs, stimulating their proliferation, migration, and tubulogenesis. This signaling cascade is closely associated with lymphatic invasion, lymph node metastasis, and poor prognosis in PDAC patients [132]. Notably, VEGF-D is another ligand in the VEGF family that also contributes to this pathway and has been implicated in enhancing lymphangiogenic activity in PDAC.

Therapeutically, multiple interventions targeting this axis have shown promise. Small-molecule inhibitors such as MAZ51 and VGX-100, as well as VEGF-C neutralizing antibodies, have demonstrated robust preclinical efficacy in reducing LVD, suppressing lymph node metastasis, and limiting tumor dissemination [133]. In orthotopic mouse models of PDAC, VEGF-C knockdown via short hairpin RNA (shRNA) or pharmacologic blockade significantly reduced micro-lymphatic vessel density (MLVD), particularly at the invasive tumor front where tumor–stromal interactions are most active [107].

Functional validation has also been provided at the mechanistic level. Recombinant VEGF-C enhances LEC migration and tube formation in vitro, while administration of a VEGFR-3/Fc chimera protein suppresses these effects [134]. Furthermore, antisense oligodeoxynucleotides (ASODNs) targeting VEGF-C decrease both lymphatic vessel formation and nodal metastasis in PDAC xenografts [135]. Altogether, these studies consolidate the VEGF-C/VEGFR-3 axis as a cornerstone of lymphangiogenesis in PDAC. They not only highlight its prognostic significance but also underscore its therapeutic potential. Future strategies may benefit from combining VEGF-C/VEGFR-3 inhibitors with systemic chemotherapy or immunotherapy to curtail early metastatic dissemination and improve patient outcomes.

### 8.2. TGF-β Signaling and Immune–Stromal Modulation of Lymphangiogenesis in PDAC

Lymphangiogenesis in PDAC is not solely driven by tumor-intrinsic factors but is also shaped by a complex network of cytokine signaling, immune cell modulation, and stromal remodeling. Among these, TGF-β signaling has emerged as another central orchestrator of the lymphatic metastatic niche.

TGF-β promotes PDAC lymphangiogenesis through multiple converging pathways. It upregulates amphiregulin, thereby activating EGFR/HER2 signaling and enhancing the expression of pro-lymphangiogenic genes. Additionally, TGF-β induces the expression of sphingosine-1-phosphate receptors (S1PR1–3), which activates downstream STAT3 and NF-κB pathways. These are both associated with lymph node priming and metastatic readiness. Importantly, TGF-β also stimulates the CCR7/CCL21 axis and thus facilitates chemokine-guided migration toward lymphatic vessels [121]. Furthermore, it modulates the tumor immune microenvironment by promoting the infiltration of regulatory T cells (Tregs), M2 macrophages, and N2 neutrophils, all of which contribute to lymphangiogenic remodeling. Combined inhibition of TGF-β receptor I and EGFR/HER2 significantly reduces tumor growth, intratumoral lymphangiogenesis, and lymphatic metastasis in orthotopic mouse models, positioning this pathway as a potent therapeutic target. Within TCGA datasets approximately 35% of PDAC tumors exhibit a combined angiogenic and lymphangiogenic gene expression signature, characterized by high levels of VEGF-C, VEGF-D, PROX1, LYVE-1, and PDPN. The KRC (Kras; Rnf43; Ptf1a-Cre) and KIC (Kras; Ink4a; Ptf1a-Cre) murine PDAC models replicated this profile, confirming their suitability for preclinical studies focused on lymphatic dissemination [121].

### 8.3. Additional Regulatory Mechanisms

Beyond canonical VEGF-C/VEGFR-3 signaling, additional regulatory mechanisms such as EV-mediated delivery of SUMOylated hnRNPA1 and epigenetic suppression of DUSP2 have been implicated in PDAC-associated lymphangiogenesis. These non-canonical pathways (discussed in detail earlier) highlight potential therapeutic opportunities targeting EV biogenesis, post-translational RNA regulation, and chromatin-modifying enzymes in KRAS-driven disease [98,104].

Further expanding the non-canonical landscape is circARFGEF2, a circular RNA specifically induced by *KRAS*^*G12D*^ and a critical mediator of lymph node metastasis. CircARFGEF2 is generated through QKI-5–dependent splicing and functions by sequestering miR-1205, thereby activating the JAK2–STAT3 axis. This promotes both lymphangiogenesis and lymphatic dissemination, introducing a novel KRAS–QKI–circRNA–STAT3 pathway that can be therapeutically exploited [136].

Transcriptomic and proteomic studies have also highlighted the WNT/β-catenin signaling axis as a driver of lymphatic spread and immune escape in PDAC, particularly in lymph node–positive (N+) tumors. WNT activation correlates with increased M2 macrophage infiltration and decreased CD8^+^ T cell activity. Inhibition of this pathway using the tankyrase inhibitor XAV-939 reduced tumor cell invasion, restored tumor suppressors such as p53 and E-cadherin, and enhanced DC activation. This suggests that WNT blockade may both suppress invasiveness and reprogram the immune microenvironment to inhibit lymphangiogenesis [114].

Neural and chemokine signaling in lymphatic spread, as noted above, is likewise important. Perineural invasion is linked to increased lymphatic remodeling, while the CXCL12/CXCR4 axis guides lymphatic-directed tumor migration, further complicating the interplay between tumor–nerve–lymphatic networks [137–139].

Together, these findings broaden the current understanding of lymphangiogenic regulation in PDAC, demonstrating that non-canonical pathways, circular RNAs, immune-epigenetic interactions, and neural signaling play essential roles in shaping the lymphatic metastatic niche.

### 8.4. Surgical Lymphadenectomy in PDAC

Lymphadenectomy is a critical aspect of the operation performed for patients with PDAC, in concert with a pancreatectomy to resect the tumor. The importance of lymphadenectomy comes from both the diagnostic and therapeutic value. The resected nodes are evaluated histologically (with hematoxylin and eosin staining), and the total number of lymph nodes positive for cancer metastases determines the N stage in the 8th edition of AJCC staging. Zero positive nodes correspond to N0, 1–3 positive nodes correspond to N1, and >3 nodes correspond to the N2 stage. This in turn can predict patient overall survival post-operatively [140]. In fact, the lymphatic stage of the disease was determined to be the most important factor in the long-term survival of patients with PDAC [141,142]. In addition to the absolute number of lymph nodes positive for cancer, the lymph node ratio is also predictive of survival. This is determined by calculating the number of positive lymph nodes out of the total number of lymph nodes resected. A ratio greater than 0.4 predicts even worse survival [143,144].

Besides the prognostic value of a lymphadenectomy, there is also data to indicate that it has therapeutic value. Specifically, the total number of lymph nodes resected surgically can impact survival. The greater the number of total nodes resected, the better the survival [145,146]. Importantly, there are factors beyond surgical technique that can affect the total lymph node yield. These include the technique of pathologic evaluation, the local hospital system, and patient factors [147,148]. It is also important to note that there are therapeutic limitations of surgical lymphadenectomy. For example, non-regional lymph nodes located around the aorta are indicative of more advanced disease [149]. While resection of these nodes can predict worse survival if they are positive for cancer, there is no evidence that more aggressive lymphadenectomy has a beneficial impact on a patient’s outcome [150]. Thus surgical lymphadenectomy is a critical aspect of patient oncologic care but must be performed in concert with the delivery of excellent systemic therapy.

## 9. Modulating Inflammatory and Immune Crosstalk in the Setting of PDAC Therapy

Tumor-associated inflammatory signals play a central role in promoting lymphangiogenesis in PDAC. Cytokines such as TGF-β1 and GM-CSF, secreted by TAMs, drive lymphatic remodeling and immune suppression. Dual inhibition of these cytokines reduces LVD and enhances the efficacy of chemotherapy, particularly gemcitabine [109].

Beyond cytokines, metabolic and transcriptional pathways also contribute to the pro-lymphangiogenic microenvironment in PDAC. As discussed previously, SPHK1–S1P signaling and the Ang2/Tie2/PI3K feedback loop enhance LEC activation via ERK and VEGFR-3 pathways, respectively. Notably, Angiopoietin-2, upregulated by TGF-β, correlates with poor prognosis and may act synergistically with VEGF signaling. These findings support the therapeutic rationale for targeting metabolic and endothelial signaling axes in combination with anti-VEGF approaches [102,119].

In contrast, several negative regulators serve as important modulators of lymphatic remodeling. PAR-2 acts as an endogenous inhibitor; its deletion enhances LYVE-1+ lymphatic vessel formation and increases lymph node metastasis [116]. Similarly, KAI1/CD82 is a metastasis suppressor that reduces VEGF-C secretion and LVD without impacting tumor growth. This suggests a selective anti-lymphangiogenic role [113]. pSTAT3, a transcription factor linked to VEGF-C upregulation, also correlates with poor clinical outcomes, positioning STAT3 inhibition as a promising strategy [115].

Adding complexity, TGF-β exhibits context-dependent duality. While it promotes lymphangiogenesis in the presence of inflammation, it can also suppress Prox1 and LYVE-1 expression in LECs. TGF-β receptor inhibition restores lymphatic sprouting, particularly when combined with VEGF-C overexpression. These results underscore the need for context-aware targeting of TGF-β signaling [118].

Hypoxia also regulates lymphangiogenesis. HIF-1α expression in PDAC correlates with increased VEGF-C, higher LVD, and more frequent lymph node metastasis. This positions HIF-1α as an upstream regulator of lymphangiogenesis in hypoxic tumor regions and a candidate target to disrupt early metastatic spread [97].

### 9.1. RNA-Based Mechanisms Driving Lymphangiogenesis in PDAC

RNA-based therapies are increasingly recognized as powerful tools for disrupting lymphangiogenesis in PDAC. EV-encapsulated miR-205–5p directly inhibits VEGFA expression in LECs. This downregulation attenuates Akt and ERK signaling, resulting in a significant decrease in lymph node metastasis. These findings establish EV-delivered microRNAs as a promising therapeutic modality for targeting pro-lymphangiogenic signaling in PDAC [151].

### 9.2. Multikinase Inhibition Targeting VEGFR and TIE Pathways

Foretinib, a multikinase inhibitor currently under clinical evaluation, potently suppresses VEGF-A, VEGF-C, and Ang-2-stimulated lymphangiogenesis in pancreatic cancer models. Mechanistically, foretinib inhibits VEGFR-2, VEGFR-3, and TIE-2 phosphorylation in LECs, disrupting both tube formation and spheroid sprouting [117]. In orthotopic and subcutaneous Panc-1 xenografts, foretinib significantly reduced tumor growth, micro-lymphatic vessel density (MLVD), and blood vessel density without significant toxicity [117]. These results suggest that multi-receptor tyrosine kinase inhibition, particularly of VEGFR and TIE pathways, may serve as an effective strategy to block both angiogenesis and lymphangiogenesis in PDAC.

### 9.3. Nanomedicine Approaches for Lymphatic-Targeted Therapy

Recent advances in nanomedicine have opened new avenues for targeting the lymphatic niche in PDAC. Lymphatic-targeted nanocarriers represent a conceptual breakthrough for enhancing drug retention in tumor-draining lymph nodes and delivering anti-lymphangiogenic payloads with improved specificity. Indeed, removal of lymph nodes may impede effective immunity. While clinical and preclinical validation is still emerging, co-delivery of anti-VEGF-C agents and cytotoxic drugs such as gemcitabine has been proposed as a promising strategy to reduce lymphangiogenesis and early metastatic spread. The modular nature of such nanoplatforms also enables co-loading of immune checkpoint inhibitors or RNA therapeutics, providing a foundation for future multi-modal lymphatic-targeted therapies in PDAC [152].

Several preclinical and translational studies have evaluated the therapeutic impact of lymphangiogenesis-targeting strategies in PDAC models (Table 5). These findings highlight both canonical and emerging pathways as actionable targets in suppressing lymphatic spread.

Together, these therapeutic strategies reflect the multifaceted role of lymphatics in PDAC progression. From molecular inhibition to nanocarrier delivery and immune reprogramming, targeting lymphangiogenesis represents a dynamic frontier with significant clinical promise.

## 10. Conclusions

The peri-pancreatic lymphatics, in concert with their control by the nervous system, play a critical role in pancreatic homeostasis and the pathophysiology of pancreatic inflammation and neoplasia. While the close relationships and pathways between lymph nodes and nerves have only started to be elucidated, significant work is left to be carried out to better understand these interactions specifically within the pancreas. For example, the specifics of sensory and sympathetic control over lymphatic trafficking and immune reactivity in pancreatic cancer are still unclear. The importance of the lymphatic system in pancreatic cancer has clearly been documented in numerous studies, showing a strong correlation between disease progression and lymphangiogenesis, immunosuppression, and lymphatic metastases. Fortunately, these pathways also provide exciting new opportunities for anatomically and biochemically targeted new therapies that are desperately needed to improve patient outcomes.

## Figures and Tables

**Table 1.** Immune cells found within the pancreas during pancreatitis.

Cells	Role in Pancreatitis	References
Neutrophilic granulocytes	Releases proteases and ROS	[79,80]
Monocytes/macrophages	Produce NFκB and proinflammatory cytokines, such as TNF, IL-6, and IL1β.	[79,81]
Pancreatic acinar cells	Release proinflammatory cytokines (TNF, IL-4, IL-6, IL-10) and chemokines (MCP-1), leading to further pancreatic damage.	[79,82,83]

**Table 2.** Transcriptomic profiling within pancreatitis.

Cell Type	Transcriptomic Profile	References
Monocytes	Increases in clusterin, oxidative phosphorylation, phagosomal signaling, apoptosis, and NF-κB signaling.	[86]
Macrophages	Increases in ribosomal signaling, oxidative phosphorylation, phagosomal signaling, MAPK pathway, apoptosis, autophagy, NF-κB signaling, and p53 pathway.	[86]
	Increases in M2 (Tgm2 and Chil3) and proinflammatory markers (Hif1a, Ccr1, and Clec4d) in Arg1+ macrophages.	[87]
	Increases in anti-inflammatory markers (Spp1 and Gpnmb) in Trem2+ macrophages.	
Neutrophils	Increases in oxidative phosphorylation, apoptosis, the PI3K-AKT pathway, and the TNF pathways.	[86]
Fibroblasts	Increases in Krt18 (AP), Smoc2 (CP), and the apoptosis, oxidative phosphorylation, p53, PI3K-AKT-mTOR, TGF-β, and TNF-α pathways.	[86]
	Increases in IL-33.	[87,88]
T helper 2	Increases in IL-4, IL-5, and IL-13.	[87]

**Table 3.** Histological Markers for Identifying Lymphatic Vessels in Pancreatic Tissue.

Marker	Cell Type/Structure Stained	Function	Specificity	References
Lymphatic Vessel Endothelial Hyaluronan Receptor 1 (LYVE-1)	Lymphatic endothelium	Hyaluronan receptor; fluid/immune trafficking	May also stain LYVE-1^+^ macrophages in inflammation	[90]
Podoplanin (also D2–40 antibody)	Lymphatic endothelium	Transmembrane glycoprotein; involved in cell adhesion and migration	Highly specific; widely used in PDAC studies	[91]
Prospero Homeobox 1 (Prox 1)	LEC nucleus	Master transcription factor for lymphatic identity	Intracellular; essential for LEC fate and maintenance	[92]
Vascular Endothelial Growth Factor Receptor 3 (VEGFR-3)	LEC membranes	Receptor for VEGF-C/D; promotes LEC proliferation and migration	Also expressed in some angiogenic blood vessels	[93]
Platelet Endothelial Cell Adhesion Molecule-1 (PECAM-1): CD31	Blood and lymphatic endothelium	Pan-endothelial marker; used to distinguish vessel types	Not lymphatic-specific alone	[94]
Hematopoietic Progenitor Cell Antigen (CD34)	Vascular endothelium	Common blood vessel marker; absent in lymphatics	Helps distinguish blood vs. lymphatic vessels	[95]
Chemokine (C-C motif) ligand 21 (CCL21)	Secreted by lymphatic endothelium	Guides CCR7^+^ dendritic cells/T-lymphocytes to lymphatics; supports transmigration	Upregulated in inflamed LECs, forms chemotactic gradients	[96]
HIF-1α	Tumor and peritumoral regions	Hypoxia-induced transcription factor; upregulates VEGF-C	Associated with increased lymphangiogenesis and metastasis	[97]

**Table 4.** Lymphangiogenic Cytokines and Molecular Regulators in PDAC.

Cytokine/Factor	Source	Mechanism	Effect on Lymphangiogenesis	References
VEGF-D	Tumor cells	Binds VEGFR-3 in LECs	Promotes LEC migration	[93]
Angiopoietin-2 (Ang-2)	LECs (stimulated by VEGF-C/TGF-β)	Activates PI3K/Akt, promotes VEGFR3 localization	Supports LEC sprouting and VEGFR3 signaling	[102,103]
TGF-β1	TAMs	Enhances fibrosis, immunosuppression, and lymphatic remodeling	Induces lymphangiogenesis and immune evasion	[109]
GM-CSF	TAMs	Stimulates myeloid differentiation and LEC activation	Promotes LVD and suppresses antitumor immunity	[109]
SPHK1/S1P	Tumor cells	Activates ERK1/2 in LECs	Increases LEC sprouting	[112]
miR-206 (↓ in PDAC)	Tumor suppressor	Suppresses KRAS and NF-κB and ↓ cytokine	Reduces VEGF-C/IL-8 levels	[111]
hnRNPA1 (SUMOylated)	*KRASG12D* mutant tumor cells	EV-mediated stabilization of PROX1 mRNA	VEGF-independent remodeling and LN metastasis	[104]
DUSP2	Tumor suppressor	Downregulation → ERK activation	↑ EV-VEGF-C	[89]
KAI1/CD82	Tumor suppressor	Downregulated VEGF-C	Inhibits lymphatic remodeling and LNM	[113]
WNT/β-catenin	Tumor cells	Regulates immune evasion and EMT	Enhances lymphatic expansion	[114]
pSTAT3	Tumor cells	Activates VEGF-C transcription	Correlation with LVD and poor prognosis	[115]
PAR-2	Tumor cells	Endogenous inhibitor	Suppresses LYVE-1+ vessel formation	[116]
Foretinib	Drug	Inhibits VEGFR-2/3, TIE-2	Blocks LEC sprouting and MLVD	[117]
miR-205-5p	Extracellular vesicles	Inhibits VEGFA in LECs	Reduces Akt/ERK activation, metastasis	[99]
TGF-β (inhibited)	Tumor cells, TAMs	LEC proliferation suppression lifted	Promotes lymphatic expansion	[118]
Exosomal lncRNA	Tumor cell-derived exosomes	Downregulation of ABHD11-AS1 enhances LEC proliferation and tube formation	Promotes lymphangiogenesis	[119]
BANCR	Tumor Cells	Sponges miR-143 → ↑HIF-1α	Upregulates VEGF-C/VEGFR-3, promotes MLVD	[120]
Amphiregulin	Tumor cells (TGF-β–induced)	Activates EGFR/HER2 → pro-lymphangiogenic gene expression	Enhances lymphatic remodeling and metastasis	[121]
S1PR1–3	Tumor cells, LECs	Activated by S1P → STAT3/NF-κB activation	Promote lymphangiogenesis and immune escape	[121]

LEC: Lymphatic Endothelial Cell.

**Table 5.** Clinical and Preclinical Studies Targeting Lymphangiogenesis in PDAC.

Model/Cohort	Findings	Conclusion	References
Patients with pancreatic head cancer (70)	High LVD at tumor margin correlates with LN metastasis, poor OS	LVD is predictive of nodal spread and prognosis	[130]
PDAC vs. benign tumors (cystadenoma, SPN)	Peritumoral LVD (not intratumoral) linked to poor differentiation, lymphatic invasion	pLVD is a strong prognostic marker	[131]
Transcriptomic profiling of PDAC tissues	High TIMP-1+ TAMs correlate with high LVD, metastasis, and poor survival	TAM-driven lymphangiogenesis is clinically relevant	[99]
Orthotopic PDAC model (ASODN against VEGF-C shRNA)	Antisense oligodeoxynucleotides (ASODNs) targeting VEGF-C reduced LVD and LN metastasis	Targeting VEGF-C is effective against lymphatic spread	[135]
Orthotopic mouse model (VEGF-C shRNA)	shRNA knockdown of VEGF-C suppressed MLVD, enhanced gemcitabine efficacy	VEGF-C inhibition enhances anti-lymphangiogenic therapy	[107]
Panc-1 xenograft model (Foretinib)	Foretinib blocked VEGC-C, Ang-2-induced MLVD; reduced tumor volume	Foretinib offers dual anti-angiogenic and lymphangiogenic action	[117]
PANC-1 in vitro + transcriptomics (XAV-939)	WNT inhibition restored immune function, suppressed migration/invasion	WNT targeting modifies immune niche and tumor invasiveness	[114]
PDAC patient cohort (VEGF-C, VEGF-D, VEGFR3 expression)	High expression levels correlated with LN metastasis and poor prognosis	Validates the VEGF-C/VEGFR-3 axis as a clinical biomarker	[100]
TCGA analysis and mouse models (angiogenic/lymphangiogenic clusters)	35% of PDACs exhibited VEGF-C/D-high, LYVE-1+, PROX1+ lymphatic signature	Suggests patient subtypes may benefit from lymphatic-targeted therapy	[121]

## Data Availability

No new data were created or analyzed in this study. Data sharing is not applicable to this article.

## Abbreviations

AP: acute pancreatitis
CGRP: calcitonin gene-related peptide
CP: chronic pancreatitis
CXCL13: CXC-chemokine ligand 13
DAMP: damage associated molecular pattern
DC: dendritic cell
DRG: dorsal root ganglion
IFN: interferon
IL: interleukin
KRAS: gene encoding K-ras protein
LEC: lymphatic endothelial cell
LN: lymph node
LTi: lymphoid tissue inducer
LVD: lymphatic vessel density
LYVE1: lymphatic vessel endothelial hyaluronan receptor 1
MCP-1: monocyte chemoattractant protein 1 (also known as CCL2)
MHC: major histocompatibility complex
MSC: myelinating Schwann cell
NE: norepinephrine
NFH: neurofilament heavy chain
NMSC: non-myelinating Schwann cell
NOD: non-obese diabetic
PAMP: pathogen associated molecular pattern
PDAC: pancreatic ductal adenocarcinoma
PNS: peripheral nervous system
RAP: recurrent acute pancreatitis
ROS: reactive oxygen species
SP: substance P
T1D: type 1 diabetes
TAM: tumor associated macrophages
TH: tyrosine hydroxylase
TLS: tertiary lymphoid structure
TME: tumor microenvironment
TNF: tumor necrosis factor
Treg: regulatory T cell
TRPV1: transient receptor potential vanilloid 1 channel
VDJC: variable, diversity, joining, contract
VEGF: vascular endothelial growth factor
VEGFR: vascular endothelial growth factor receptor
VLRs: variable-like regions
